# Letter to the Editor: No immunophenotyping in peripheral blood of prostate cancer patients treated with neoadjuvant Rituximab?

**DOI:** 10.1186/s12967-020-02496-5

**Published:** 2020-09-01

**Authors:** Paulius Bosas, Gintaras Zaleskis, Vita Pasukoniene, Feliksas Jankevicius

**Affiliations:** 1 Medical Faculty, Vilnius University, Vilnius, Lithuania; 2 Laboratory of Immunology, National Cancer Institute, P. Baublio 3b, Vilnius, Lithuania; 3 Vilnius Gediminas Technical University, Vilnius, Lithuania

Dear Editor,

The paper by Ryan et al. [1] in your recent issue addresses intriguing data on Rituximab (Rx) immunomodulation in prostate cancer (PCa) neoadjuvant setting. The report on the first 8 participants from author’s trial (NCT01804712) is appreciated since the complexity of similar studies quite often results in a termination or withdrawal. However, we would like to point out some downsides in the above-mentioned publication. The cornerstone statement that “…high B cell density is associated with biochemical failure*”* refers to the author’s own previous publication [2]. However, this reference has nothing to do with biochemical failure. Even the higher density of B cells in recurrent tumors cannot be considered a surrogate parameter of biochemical failure. Growing tumors can passively induce recruitment of immune cells into the prostate microenvironment as a result of concomitant inflammation [3]. The relationship between tumor infiltrating cells and clinical outcomes for PCa is still debatable. The authors concluded that Rx treatment applied prior to prostatectomy reduced tumor infiltration density of B and T-cells. However, Rx is well known to specifically deplete a T cell subset which also co-express CD20+ [4, 5]. As a matter of fact, the double positive CD20+CD3+ lymphocyte was demonstrated to be a specific marker of therapeutic response in rheumatoid arthritis patients receiving Rx [5]. Isn’t it possible that similar mechanisms were involved in PCa patients treated with Rx? The statement that there was a demonstration of “the inter-dependence between B and T-cells in prostate cancer…” is also questionable since the authors do not provide any evidence of double positive CD20+CD3+ lymphocyte absence in tumorous tissue. One can notice that CD20+ B cell and CD 3+ T cell infiltration scores in tumor tissue provided in Tables 2 and 3 of the paper are almost identical. This raises the question if an alternative explanation of the phenomenon might also be valid. Could it be that some specific B cell phenotype conversions or infiltration patterns were induced by Rx treatment? Unexpectedly, the peripheral blood T and B subset monitoring were not reported in this paper, although the peripheral blood B cell enumeration was originally planned as a secondary outcome measure (NCT01804712). Four out of 8 patients (Fig. 3) exhibited PSA rise while being on Rx treatment for only 29 days (one patient displayed a steep 40% PSA increase). We would suggest adhering to weekly PSA monitoring in the future if this study will continue. Rx was shown to induce paradoxical IgM and serum viscosity increase in some hematological malignancies [6], leading to increased morbidity. Therefore, it might be premature to rule out the fact that Rx treatment could occasionally provoke PCa progression. Unfortunately, the PSA data on day 29 of the control group participants was not provided. This would have been helpful to understand if some PSA flares in the interventional group were merely accidental (laboratory error?).

This letter is not intended to provide a critique of an interesting study. We only want to draw attention to the complexity of Rx effects on therapeutic B cell modulation. Also, we would like to point out the necessity to explore extensive lymphocyte phenotyping in blood once patients are treated with B lymphocyte depleting drug.

## Data Availability

Not applicable.
